# Perceived Causal Networks: Clinical Utility Evaluated by Therapists and Patients

**DOI:** 10.17505/jpor.2023.25260

**Published:** 2023-06-17

**Authors:** M. Andreasson, J. Schenström, J. Bjureberg, L. Klintwall

**Affiliations:** 1 Department of Psychology. Uppsala University, Sweden; 2 Centre for Psychiatry Research, Department of Clinical Neuroscience, Karolinska Institutet & Stockholm Health Care Services, Region Stockholm, Sweden

**Keywords:** Network theory of mental disorders, case conceptualization, depression, comorbidity, perceived causal relations

## Abstract

Conceptualizing psychiatric disorders as idiosyncratic networks of mutually reinforcing behaviors and emotions has a long history in the form of psychotherapy case conceptualizations created collaboratively by therapist and patient. However, such methods are typically unsystematic and biased by therapist assumptions. An alternative is Perceived Causal Networks (PECAN), a structured online questionnaire in which patients quantify causal relations between problematic behaviors and emotions, and responses are visualized in the form of a network. In the present study, PECAN was evaluated for clinical utility at the start of therapy for five patients screening positive for depression. As expected, the five networks were found to be highly idiosyncratic, with two revealing the expected maintaining feedback loops. Both therapists and patients evaluated the method as useful in the initial phase of a therapy treatment. Although PECAN shows promise as a clinical tool, findings suggest that the method could be improved by including contextual factors maintaining depression.

## Introduction

The network theory of mental disorders proposes that psychiatric symptoms are partially maintained by causing each other, and that this might be idiosyncratic across patients (Borsboom, 2017). This challenges the conventional common-cause view, exemplified by the DSM-V (American Psychiatric Association, 2013) where symptoms are tacitly seen as indicators of an underlying disease. Using the example of depression, the conventional view would thus be that a patient presenting with poor sleep, anhedonia, feelings of worthlessness, and at least two more symptoms indicate the presence of depression, and that those symptoms are caused by the depression. Critics of the conventional view argue that the high rate of comorbidity between different psychiatric disorders, the failure of research to identify consistent etiologies, and the non-specific nature of most treatments, show that this research paradigm has failed (Cramer et al., 2010).

Network theory would in contrast propose that poor quality of sleep may cause anhedonia, which may in turn cause feelings of worthlessness and so on. Importantly, network theory also suggests that when such symptom-to-symptom causation results in feedback loops (e.g., sleep problems causing anhedonia which in turn causes feelings of worthlessness which then feeds back into sleep problems), this might explain why a pathological state such as depression is maintained over time, even when the life-event triggering the episode (e.g., a newborn baby causing sleep problems) is no longer present (McNally, 2016). The symptom relations in network theory are graphically represented in symptom-networks where symptoms are shown as “nodes” and causal inter-symptom relations are shown as “edges”.

It is worth noting that although these networks are typically called “symptom networks”, the nodes in the network can in principle be anything, from contextual factors (e.g., financial problems) and somatic illness (e.g., cancer) to problematic behaviors and emotions typically not seen as symptoms (e.g., procrastinating homework). Nodes can be seen as the conscious complaints a patient might come into treatment with, which can be contrasted with edges - the processes between nodes. These processes can be biological (e.g., insomnia causing fatigue), psychological (e.g., conflicts causing rumination) or be part of a social context (e.g., procrastinating causing conflicts with parents), and they can be invariant across individuals (e.g., everyone has the edge between insomnia and fatigue) or variable across individuals (only for some people, conflicts cause rumination) and even within individuals across time (only during school weeks does procrastination cause conflicts with parents). The specific mechanism or process behind an edge can be known or unknown. Potentially, several edges can share the same underlying mechanism (e.g., the edge from anxiety to drug-use and the edge from anxiety to self-harm both constitute emotion regulation/negative reinforcement).

Whereas the severity of a psychiatric disorder in the conventional view is thought to be a function of the strength of the underlying entity (Borsboom et al., 2016), network theory propose that the severity of the disorder is a function of the strength of the causal connections between symptoms, and whether these connections form feedback-loops that will maintain the disorder across time and make the network stable even when perturbed by interventions.

### Case Conceptualizations

In psychotherapy, case conceptualizations are idiographic, typically collaborative hypotheses used to identify functional relations between the patient’s problems, goals, and factors influencing them (Haynes et al., 2018). The content of a conceptualization is dependent on the therapists’ theoretical allegiance and the patients’ presenting problems, but is a prerequisite for planning psychological treatment (Easden & Kazantis, 2018). For a conceptualization to be useful in treatment, it must identify modifiable processes that maintain symptoms. A well-formulated case conceptualization should improve patient motivation and help patients to talk constructively about the problems. Therapists use a variety of scientifically validated methods and instruments to form a complete conceptualization (Haynes, 2020). However, the validity of these conceptualizations is typically low, likely due to therapist biases (Meehl, 1954; Haynes et al., 2018).

### Data-Driven Case Conceptualizations

Ecological Momentary Assessment (EMA; Shiffman et al., 2008) is a method that has been used to create formalized and idiographic case conceptualizations (Fisher et al., 2017; Frumkin et al., 2021). EMA works by prompting a respondent a few times daily (typically 5 to 10 times) to report which symptoms are experienced since the last prompt. Data are collected in this manner for a few weeks, until a statistical analysis on the level of the individual can be conducted. Simply put, time-series analysis detects temporal patterns between symptoms, for example by looking at how symptoms predict changes in other symptoms to the next time-point. The network can then be analyzed for centrality of nodes or edges (i.e., the importance of that node or edge in maintaining the network, indicating its potential as a treatment target), or even be used to run simulations of specific interventions targeting different symptoms (Lunansky et al., 2022).

Several studies have attempted to apply EMA to construct symptom networks in clinical settings, with promising results (Levinson et al., 2021, Levinson et al., 2023, Frumkin et al 2021, Soyster et al., 2019). However, limitations of networks created using EMA must be noted. First, as the assessments are conducted with a specified interval (typically a few hours), changes that occur in a shorter time frame (e.g., rumination causing sadness) cannot be identified. Causation at such short time-frames can be captured by augmenting the directed network with a contemporaneous network, showing which symptoms tend to co-occur at the same time points. However, such analysis does not reveal the causal direction. Conversely, long-term effects, such as physical inactivity causing fatigue over several weeks, will also not be identified. Finally, symptoms causing another symptom through avoidance (e.g., isolating socially to avoid anxiety) cannot be detected, since the tentative symptoms being avoided are not necessarily experienced.

### Perceived Causal Problem Networks

An alternative to EMA-methods, but with the same goal of creating idiosyncratic case conceptualizations in the form of networks, is to rely on self-reported causality between symptoms, a method called Perceived Causal Relations (PCR; Frewen et al., 2012; Frewen et al., 2013). The method is based on the observation that “individuals tend to perceive their behavior, life situations, and psychological and physical symptoms as causally related events (Frewen et al., 2012). Although such causal attributions may deviate from actual causal relations, they are of interest to measure in and of their own right, revealing how participants’ think about themselves and their problems (Frewen et al., 2012).

An updated method, Perceived Causal problem Networks (PECAN) was specifically developed as a clinically useful alternative to EMA-methods (Klintwall et al., 2023). The PECAN questionnaire is composed of four steps. First, the respondent is presented with the list of problematic behaviors and emotions used in Klintwall et al. (2023). The respondent is asked to select those of the items experienced “almost every day in the past two weeks”. Next, the respondent is asked to specify each selected problem in his/her own words, in order to individualize the questionnaire and help the respondent commit to a specific understanding of the item. Then, the respondent is asked to rate the severity of each selected problem, on a scale from 0 to 100. Finally and most importantly, the respondent is presented with each selected item and asked to select which of the other problems were the perceived causes for this particular problem. For the items selected as causes, the respondent is then asked to distribute the causal strength of each selected causal item, and the option “don´t know/other causes”, so that the causal strengths sum to 100. These percentages then constitute the edges (i.e., causal inter-symptom directed relations) in the resulting visualized network, placing nodes (i.e., symptoms/problems) according to a force-directed algorithm. Node size typically denotes problem severity.

The aim of PECAN is to measure the perception of causal relations. These perceptions are seen as containing useful information for conceptualizations in their own right and pragmatically within reach, given the current shortcomings of time-series analysis. Furthermore, patient’s causal attributions are known to predict psychological adjustment, behaviour and compliance in the somatic health domain (Brogan & Hevey, 2009). This adheres to Borsboom’s concept of validity stating “A test is valid for measuring an attribute if (a) the attribute exists and (b) variations in the attribute causally produce variation in the measurement outcomes” (Borsboom, 2004). PECAN strives to accurately measure individual perceptions of inter-symptom causal relations. This puts it in contrast to EMA which strives to measure true causal relations by assuming time-series correlations as implicating true processes of inter-symptom causality. PECAN, however, is not validated or otherwise tested pertaining to the degree in which it corresponds to other measurements of causal relations derived from experimental analysis.

Klintwall and colleagues (2023) tested PECAN with a sample of adults screening positive for depression, recruited from social media (*N* = 231) and found that the method was fairly fast (median completion time 22.7 minutes), and showed acceptable immediate test-retest reliability for node centrality (*r* = .81, *SD* = .14). Rated by therapists (*n* = 50), the networks were judged to be logical (57 %), show identifiable treatment targets (66 %) and to explain the maintenance of the patients’ depression (55 %). According to the participating therapists, the presented networks would on average correspond to roughly half of the assessment phase of a typical therapy. Nine out of ten reported that the method was promising as a basis for discussion together with a client.

These evaluations were based on networks reported by participants who were recruited from social media, answering PECAN unrelated to any therapy. The therapists scoring them did so after a quick review, and without knowing the patients. While these findings are interesting, they also raise further questions about the potential clinical utility of PECAN. The purpose of the current study was to survey patients' and therapists’ evaluations of the clinical utility of symptom-networks created with PECAN in a psychotherapy setting.

## Method

### Participants

The study included five therapist-patient dyads. The therapists were recruited through convenience sampling in Sweden, through psychotherapist email lists, social media and through collegial recruitment. The therapists were all trained in cognitive behavioral therapy, and varied in experience from being recently licensed to having over two decades of practice. Of the 23 therapists who agreed to be contacted regarding the study, 12 therapists chose to participate in the study. These therapists recruited patients in their routine clinical practice and were instructed to include patients with depressive and/or anxious symptoms, although a formal diagnosis was not required for including a patient. No financial or other compensation was given to patients or therapists.

Inclusion criteria for patients were as follows: (1) age above 16 years; (2) five or more selected items in PECAN; and (3) completion of the PECAN questionnaire. Exclusion criteria were (1) verbally reporting not having answered the PECAN questionnaire carefully; (2) a diagnosis of psychosis or bipolar disorder; (3) treatment not continued past the initial session; and (4) incomplete answers on follow-up questionnaires.

Therapists contributed with at least one patient each, adding up to 14 participating therapist-patient couples. Due to incomplete questionnaires (the last exclusion criterion), nine patients were excluded: six patients failed to answer the patient evaluation questionnaire, and three of the therapists failed to answer one or both of the therapist evaluation questionnaires. Thus, the number of therapist/patient couples participating in the study was five (see Table 1). The names of the participants are pseudonyms.

**Table 1:** Demographics of the participating patients.

Patient	Age	Gender	Duration of psychological distress	PHQ-9 score
Alba	31-40 yrs	Female	6-12 months	19
Betty	21-30 yrs	Female	<1 month	17
Carl	31-40 yrs	Male	>12 months	6
Dora	41-50 yrs	Female	> 5years	18
Eve	51-60 yrs	Female	> 12months	12

*Note*. Ages are reported in ranges to ensure anonymity. The names are pseudonyms.

### Procedure

At first contact with a patient that the therapist deemed suitable for inclusion, the patient was invited to participate in the study. Participation began with the patient receiving information about the study and giving their written consent. This was followed by the patient answering the online PECAN questionnaire at home, which was reached by a link provided by the therapist.

In the next therapy session, the patient and the therapist together viewed the patient's idiosyncratic network. Therapists were free to select any interventions they deemed suitable for the patient, regardless of what the symptom network might suggest. Two evaluations of the network were made by the patient and therapist: the Patient Questionnaire (PQ) and the Therapist Questionnaire-1 (TQ1). Three to five sessions later, when the therapists deemed themselves relatively sure of their conceptualization of the patient, the therapist answered a second questionnaire (TQ2). These three questionnaires are described in detail below. The study was approved by the Swedish Ethical Review Authority (ID 2019-06410).

### Materials

#### Perceived Causal Symptom Networks

As described above, PECAN is an online questionnaire which first lets participants select relevant problematic behaviors and emotions from a list of 26 common problematic behaviors and emotions, and then rate the causal links between the selected items. This was done between sessions, without the therapist. As with the previous version of PECAN, emotions were not available as causes to other emotions. In communication with patients, the networks were referred to as problem maps. The visualization was interactive, so that the respondent could move nodes around and select different cut-offs for how strong edges had to be in order to be shown (the default for this being set so that the number of edges shown did not exceed the number of nodes).

#### Patient Health Questionnaire-9

The Patient Health Questionnaire (PHQ-9; Kroenke et al., 2001) was used to confirm the presence of depressive symptoms. The questionnaire measures the experiencing of the nine criteria of major depressive disorder the last two weeks on a frequency scale ranging from 0 (Not at all) to 3 (Nearly every day) and gives a total score summing all items. PHQ-9 has been validated in at least 27 studies, averaging a sensitivity of 0.77 and a specificity of 0.85 using a cut-off score of 10 (Manea et al., 2015). It shows good reliability and internal consistency (Sun et al., 2020)

#### Questionnaires Measuring Experienced Clinical Utility

To measure the patients’ and therapists’ experience of clinical utility of PECAN as an assessment tool, three questionnaires, the Patient Questionnaire (PQ), the Therapist Questionnaire-1 (TQ-1), and the Therapist Questionnaire-2 (TQ2), were developed for the study. Neither of these questionnaires were tested for validity or reliability before being used in the present study. The items in the questionnaires were based on the themes identified by Redhead and colleagues (2015) of clients’ experiences of conceptualizations in cognitive behavior therapy, and with regard to properties and elements that are considered to be of relevance in assessment (Bieling & Kuyken, 2003; Flitcroft, 2007; Kuyken et al., 2016; Easden & Kazantzis, 2018).

The Patient Questionnaire (PQ) consists of six items measuring different aspects of clinical utility as perceived by patients. It was to be answered by the patient at start of treatment. Items are statements answered on a five-point Likert scale from 1 to 5 (1: “not correct at all”, 2: “slightly correct”, 3: “partly correct”, 4: “fairly correct”, 5: “completely correct”).

The Therapist Questionnaire-1 (TQ-1) was to be answered by the therapist at the start of treatment, and the Therapist Questionnaire-2 (TQ-2) was to be answered by the therapist 3-5 sessions into the treatment. The TQ1 and TQ2 both measure clinical utility but differ in that TQ1 asks about the expected utility of the tool, whereas TQ2 evaluates the method when the conceptualization phase of therapy is finished. Items are statements answered on a five-point Likert scale from 1 to 5 (1: “not correct at all”, 2: “slightly correct”, 3: “partly correct”, 4: “fairly correct”, 5: “completely correct”). All of the questionnaires were originally created in Swedish.

### Analysis

Individual networks and evaluation ratings are presented for the five participants, with averages across items for each questionnaire for that therapist or patient. Averaging across participants was not deemed useful, given the small sample size.

## Results

Table 2 describes the five patients’ responses to the Patient Questionnaire (PQ), whereas Table 3 describes the therapists’ responses to TQ-1 and TQ-2.

**Table 2:** Responses on the Patient Questionnaire (answered on a scale 1-5)

	Alba	Betty	Carl	Dora	Eve
“The problem map shows how my problems actually are related”	4	5	4	4	4
“The problem map helped me to better understand my problems”	4	3	5	4	4
“The problem map made me more motivated to change”	3	3	3	5	5
“To answer the questionnaire or to see the problem map made me experience distress”	1	2	1	3	1
“The problem map made it easier for me to talk about my problems”	1	3	3	2	3
“The problem map shows what I should change to feel better”	3	5	5	4	4

**Table 3:** Responses on Therapist Questionnaire 1 (TQ1) and Therapist Questionnaire 2 (TQ2) (answered on a scale 1-5).

	Alba’s therapist		Betty’s therapist		Carl’s therapist		Dora’s therapist		Eve’s therapist	
	TQ1	TQ2	TQ1	TQ2	TQ1	TQ2	TQ1	TQ2	TQ1	TQ2
“The problem map will be useful to me as a therapist”	4	4	5	3	5	4	5	4	5	5
“The problem map helps me make decisions about the treatment”	4	3	4	3	5	4	5	3	5	5
“The problem map explains how the problems are maintained”	3	4	4	3	5	4	4	5	5	5
“The problem map includes the patient's most important problems”	3	3	5	4	4	3	5	5	5	5
“The problem map adds something important beyond what I usually do”	3	3	4	3	4	5	2	3	4	5
“The problem map’s causal arrows correspond with my current analysis”	2	3	3	3	4	4	3	4	5	5
“The problem map caused the therapy to end up on the wrong track (e.g. working with the wrong problem)”	-	1	-	2	-	1	-	1	-	1
Minutes of session-time saved using PECAN	0	-	30	-	120	-	0	-	120	-

*Note*. The items in the second questionnaire (TQ2) were posed in the past tense. For example, the sixth item in TQ2 was expressed as “The causal arrows problem map’s corresponded with my current analysis”.

### Alba

**Figure 1:**
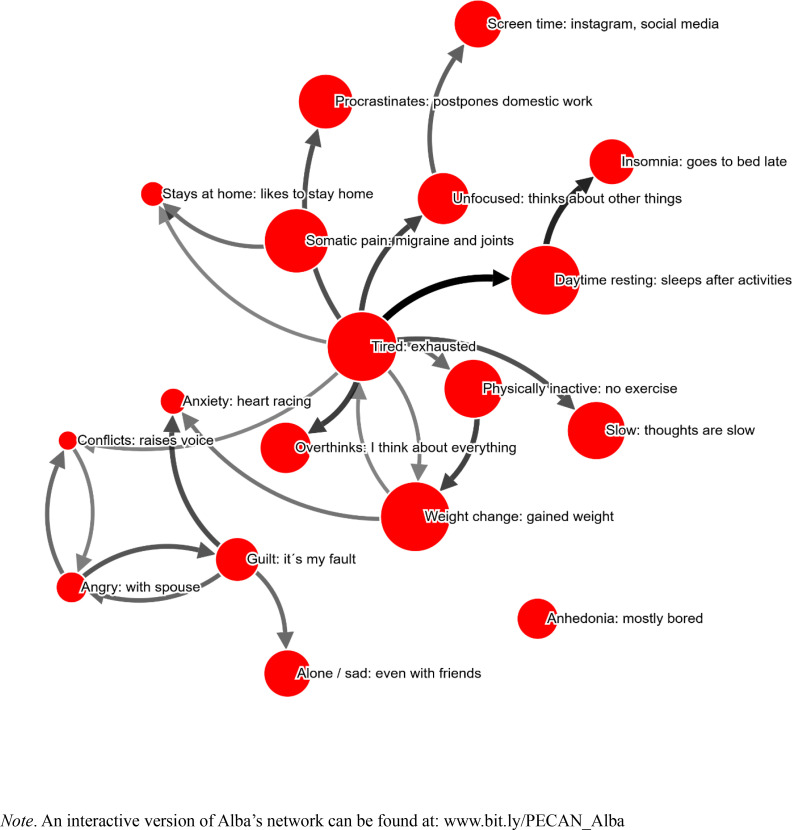
Alba’s network. Node size corresponds to self-reported severity of the problem. Arrow width/color indicates strength of perceived causality. Only edges stronger than 30 % shown.

It took Alba 35 minutes to complete the PECAN, and 18 symptoms were selected (see Figure 1). Symptoms were on average perceived to be 82 % caused by other symptoms (as opposed to “Don´t know/other causes”).

“Tired: exhausted” is the most central symptom in this network. Based on visual analysis, the network seems to be maintained by a central feedback loop between “Physically inactive: no exercise”, “Weight change: gained weight”, and “Tired: exhausted”. Through “Tired”, this loop maintains the majority of symptoms in the network, aside from a smaller cluster of symptoms which in turn is maintained by a loop between “Guilt” and “Angry”. Note that the cutoff of this visualization is 30 %, meaning that weaker relations are not shown.

The therapist reported that time was neither lost nor saved by completing and reviewing the network. The therapist commented at TQ1 that “PTSD is missing”. When asked whether the symptom network added something beyond what is usually done in therapy, the therapist commented that “the patient has to think over how [problems] are connected”. Given this perceived causal network, it would make sense to intervene by breaking the central feedback-loop between “Physical inactivity”, “Weight gain”, and “Tired”. This could be done by behavioral activation, increased exercise, or a combination of the two.

### Betty

**Figure 2:**
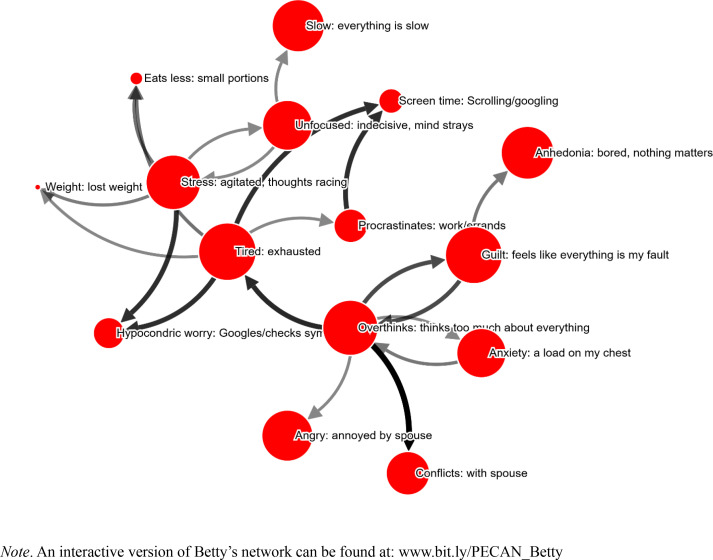
Betty’s network. Node size corresponds to self-reported severity of the problem. Arrow width/color indicates strength of perceived causality. Only edges stronger than 34 % shown.

It took Betty 27.6 minutes to complete the PECAN, and 15 symptoms were selected (see Figure 2). Symptoms were on average perceived to be 95 % caused by other symptoms (as opposed to “Don´t know/other causes”).

As in the previous network, “Tired: exhausted” is the most central symptom in this network and the perceived cause of a host of symptoms. But it differs from the previous network in the relations between these symptoms. Most importantly, there are no central feedback loops, that is, no symptom caused by tiredness is reported to have a strong causal influence going back to tiredness. That said, the symptom “Overthinks: thinks too much about everything” has a strong causal influence on the tiredness and is in itself subject to feedback loops: it is both the cause and result of the symptom “Guilt: feels like everything is my fault” and “Anxiety: a load on my chest”. One interpretation is that overthinking is maintaining tiredness, while the overthinking itself is being maintained through guilt and anxiety. While filling out PECAN, Betty commented that it was “interesting and rewarding”.

The therapist commented that “it is good that the patient herself had to think before the first visit of how the symptoms are connected”, presumably meaning that the patient is prompted to think about their symptoms in a causal-relational manner.

### Carl

**Figure 3:**
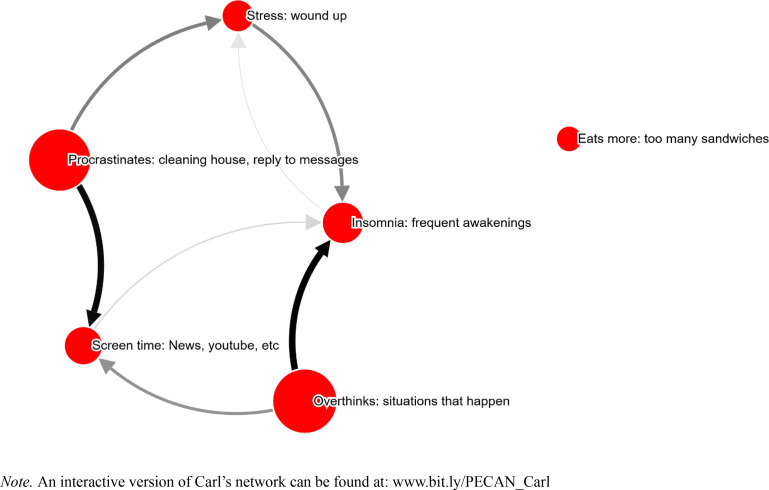
Carl’s network. Node size corresponds to self-reported severity of the problem. Arrow width/color indicates strength of perceived causality. All edges shown.

It took Carl 21.9 minutes to complete the PECAN, and six symptoms were selected (see Figure 3). Symptoms were on average perceived to be 37 % caused by other symptoms (as opposed to “Don´t know/other causes”).

No feedback loop between the symptoms can be identified, which may represent an actual absence of such. The lack of a feedback loop may also be due to a patient's low understanding of the relations between symptoms, or that the network is missing relevant nodes, possibly contextual nodes.

The therapist commented at TQ1 that “the patient participates from the start, becomes more active and can talk about the connections himself before I try to illustrate via functional analysis”, also adding that the network is a “good visual illustration”. At TQ2, the therapist also added that the network as a conceptualization added “increased clarity with the help of images, not just functional analyses”.

### Dora

**Figure 4:**
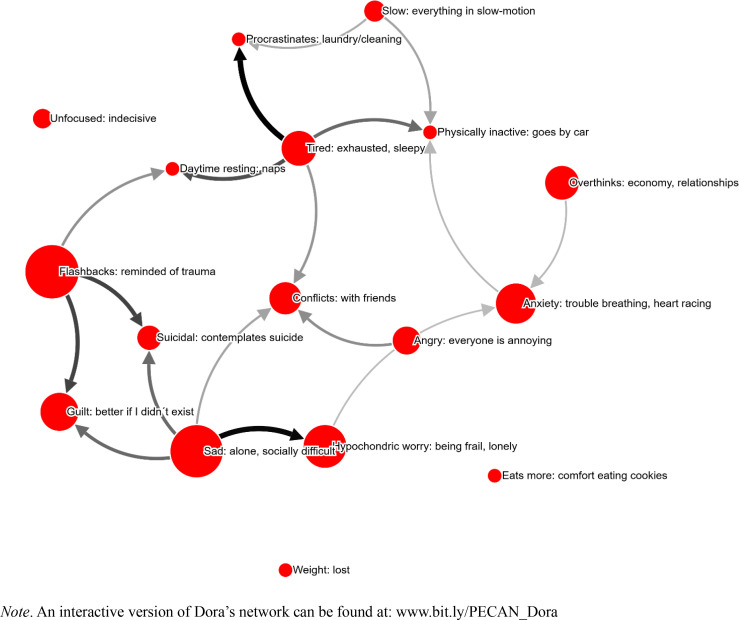
Dora’s network. Node size corresponds to self-reported severity of the problem. Arrow width/color indicates strength of perceived causality. All edges shown.

It took Dora 45.9 minutes to complete the PECAN, and 17 symptoms were selected (see Figure 4). Symptoms were on average perceived to be 42 % caused by other symptoms (as opposed to “Don´t know/other causes”).

When given the option to give the cause of the problem “Flashbacks”, she answered that her “ex [partner] found out [her] address”. The interpretation here is that her past abuser knows of her place of residence, which likely would be a major cause of distress and psychological symptoms, PTSD being the most prominent one. If this is the case, it could be argued that Dora’s situation at the time is not mainly dominated by a psychiatric problem, but rather caused by the threat of her aggressor. It would be likely that Dora’s needs are mainly that of security and support – not primarily psychotherapy.

Dora had to fill out PECAN in session due to external events, making it hard for the therapist to judge if time was saved. The therapist commented that PECAN was a “pedagogical way of creating a conceptualization of the patient”, and that they also thought that “it can be very motivating” (TQ1). Later on, in TQ2, she commented that PECAN is “a more comprehensive conceptualization of symptoms, which became validating and motivating for the patient”.

### Eve

**Figure 5:**
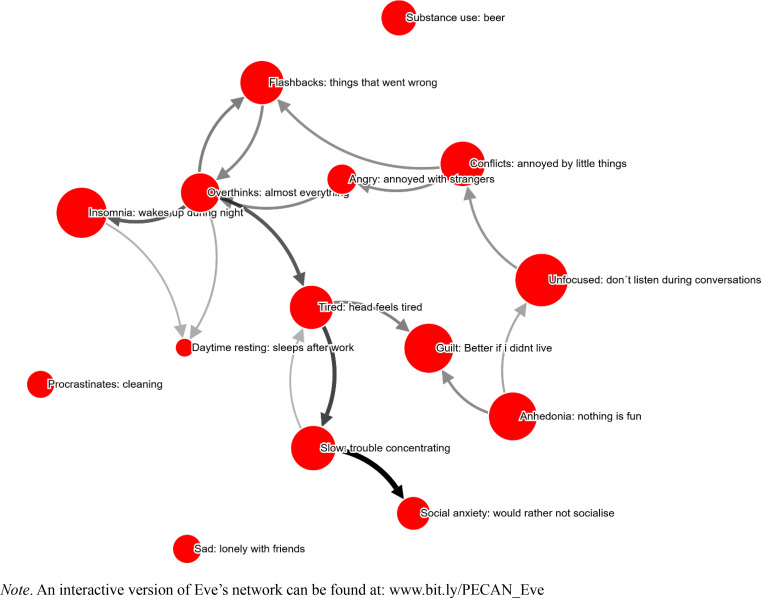
Eve’s network. Node size corresponds to self-reported severity of the problem. Arrow width/color indicates strength of perceived causality. Only edges stronger than 34 % shown.

It took Eve 64.1 minutes to complete the PECAN, and 15 symptoms were selected (see Figure 5). Symptoms were on average perceived to be 70 % caused by other symptoms (as opposed to “Don´t know/other causes”).

While this network lacks a central feedback loop, there seems to be a self-maintaining causal relation between “Overthinks: almost everything” and “Flashbacks: things that went wrong”. The overthinking, in turn, causes a number of symptoms directly (“Insomnia: wakes up during night”; “Day time resting: sleeps after work”; “Tired: head feels tired”) and indirectly (“Slow: trouble concentrating”; “Social anxiety: would rather not socialize”; “Guilt: better if I didn’t live”). After answering the questionnaire, the patient commented that it was “challenging, illogical at times”. The therapist commented that it had been necessary to “explain what the map showed and what the idea of the arrows are”. At TQ1, the therapist commented that the network was a “good visual illustration that quickly makes the client involved and positively surprised about how problems are connected and how to influence their situation”.

## Discussion

Unsurprisingly, patients reported the visualized networks to correspond to their own views of how their problems were related. They also reported that seeing the network improved understanding of problems, increased motivation for change, and indicated what could be done to improve their state. To a lesser extent, patients also reported that the network increased their ability to talk about their problems. Importantly, seeing the network did not lead to distress in patients.

Therapists reported that the idiosyncratic network was beneficial to therapy, that it was helpful in treatment decisions, showed how patient problems were maintained, and to a lesser extent, included the most important problems the patient experienced, added something novel to treatment, and corresponded to the therapists’ own conceptualization of the patient.

The networks show a variety of different symptom-patterns, representing the heterogeneity within a clinical population. This is in line with the network paradigm of psychopathology and shows the need for idiosyncratic case conceptualizations.

Low-functioning patients reported feeling overwhelmed by the questionnaire and rendered symptom networks that included so many nodes that it might be hard to make sense of the network. Future versions of PECAN might ask the respondent to prioritize the nodes to include in the network. In many cases external contextual factors played a central role in the patients’ perception of their problems, but contextual nodes were not included in the present version of PECAN, an issue that could be resolved in future versions.

Patient feedback indicated that the estimation of causal strength using percentages was not very intuitive, and that the notion of causal strength itself proved difficult for patients to grasp. This last point could be addressed by quantifying edges differently (e.g., “how often does X lead to Y?” or “How certain are you that X is a cause of Y?”). This last point might be of special interest, as it opens the possibility to combine a generic psychopathology model, based on clinician knowledge, with idiosyncratic perceived relations similar to PECAN.

We replicated the finding that patients perceive symptoms to be causally related to one-another (Frewen et al., 2012), as network theory also posits. However, this does not indicate validity of idiographic individual networks as a method to create case conceptualizations. That being the case, patient perceptions of causal relations are in itself clinically useful, regardless of the truthfulness of the causal relations. Cognitive structures such as thoughts, rules, defense mechanisms etc. are important phenomena in most schools of psychotherapy, and usually form part of clinical conceptualizations and treatment plans. This can be illustrated by the patient Alba in our sample, who perceived tiredness as a cause to many of her other problems. This perception is clinically meaningful, while simultaneously perhaps being ontologically false. Informed by PECAN, an intervention targeting the perception itself could form part of a treatment. For example, changing the assumption that negative emotions cause behaviors and not the other way around is one of the treatment targets in behavioral activation for depression (Martell et al., 2021), thereby modifying a perception of causality that likely maintains the experienced problems.

It must be noted that our sample of patients is not representative of any clinical population or setting due to the convenience sampling recruitment process and small sample size. The study probably suffered from a systematic dropout of patient-therapist dyads who disliked the tool initially and therefore did not complete the questionnaires, possibly leaving the more enthusiastic participants in the sample. Another major limitation is the unknown reliability and validity of the networks. Patients likely have trouble remembering both what problems they typically experience, and probably have even more trouble recalling or hypothesizing the perceived causes of these problems (for an alternative to PECAN, which uses repeated brief daily assessments of perceived causes to create networks, see Burger et al, 2022). Similarly, it is easy to imagine that some edges are consistently unreliable (e.g., causes for worrying), and others are probably consistently under-reported (e.g., lack of physical activity leading to fatigue) or overreported (e.g., insomnia leading to trouble focusing the day after).

The method could be improved by enabling the removal, addition, and modification of nodes and edges on the symptom network after having seen the resulting network. This would allow for a more collaborative process, using the symptom network as a living conceptualization, rather than a fixed product. The symptom network would also be more relevant throughout therapy, as changes in the patients’ functioning could be represented in the symptom network.

This is a proof-of-concept study, showing the feasibility of network-based self-assessment instruments for clinical conceptualization. This version of PECAN, including causal question phrasings or the item-list used, should not be considered definite. The therapist questionnaire in the current study contained one item measuring the validity of the conceptualization, as compared to the therapists’ own analysis a few sessions into treatment (”The problem map’s causal arrows correspond with my current analysis”), showing medium-high scores. We also measured client face-validity through the item “The problem map shows how my problems actually are related” rendering high scores. These indicators of validity show promise, but do not suffice to deem the tool validated. Future versions should be psychometrically validated, ideally through empirical-analytical methods such as those described in Mumma and Fluck (2016) and through measures of correspondent validity with gold-standard measurement such as formalized clinician-generated case conceptualizations. Further tests of reliability in clinical settings are also warranted in order for future versions of PECAN or other network-based self-assessment instruments to be fit for standard practice.

## Action editor

Lars-Gunnar Lundh served as action editor for this article.

## Open access

This article is distributed under the terms of the Creative Commons Attribution 4.0 International License (http://creativecommons.org/licenses/by/4.0/), which permits unrestricted use, distribution, and reproduction in any medium, provided you give appropriate credit to the original author(s) and the source, provide a link to the Creative Commons license, and indicate if changes were made.
